# Supplementary material for: Targeting the Structural Integrity of Extracellular Vesicles via Nano Electrospray Gas-Phase Electrophoretic Mobility Molecular Analysis (nES GEMMA)
Source: Membranes (Basel). 2022 Sep 9;12(9):872. doi: 10.3390/membranes12090872 (PMC9501092; doi:10.3390/membranes12090872)
Supplement: Supplementary file 1 [file membranes-12-00872-s001.zip › membranes-1836033-supplementary.pdf]

membranes

Supplementary Materials

Targeting the Structural Integrity of Extracellular Vesicles via Nano Electrospray Gas-Phase Electrophoretic Mobility Molecular Analysis (nES GEMMA)

Stephanie Steinberger [1], Sobha Karuthedom George [2], Lucia Lauková [2], René Weiss [2], Carla Tripisciano [2], Martina Marchetti-Deschmann [1], Viktoria Weber [2], Günter Allmaier [1] and Victor U. Weiss [1,*]

[1] Institute of Chemical Technologies and Analytics, TU Wien, 1060 Vienna, Austria
[2] Center for Biomedical Technology, Department for Biomedical Research, University for Continuing Education Krems, 3500 Krems, Austria
* Correspondence: victor.weiss@tuwien.ac.at; Tel: +43-1588-0115-1611

The supplementary information gives a flow cytometric protocol to characterize EVs from different cellular origin (Figure S1), controls for the flow cytometric characterization of EVs (Figure S2), data on the impact of varying sheath flow values in the nDMA on recorded signals (Figure S3) as well as data on fitting of Gaussian peaks to recorded spectra (Figure S4 as well as Table S1).

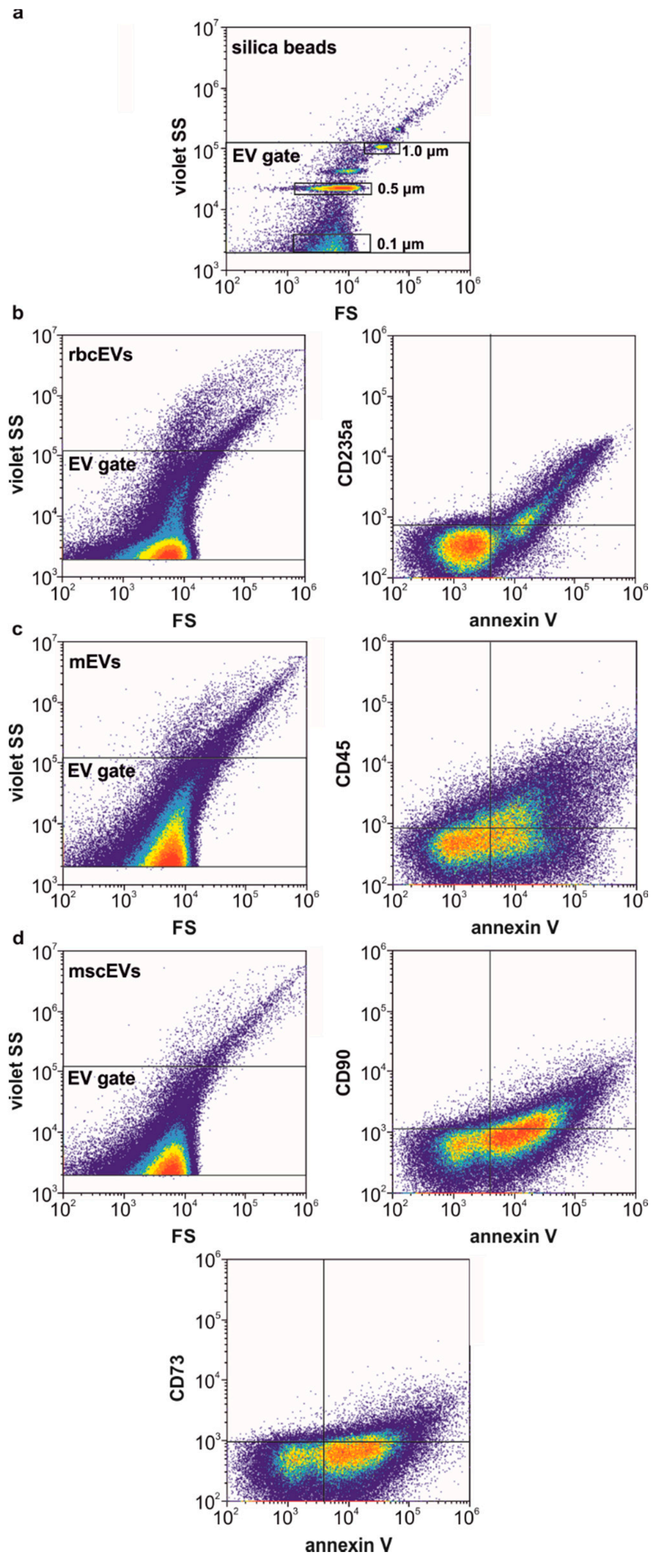

Figure S1. Flow cytometric protocol to characterize EVs from different cellular origin. (a) Flow cytometric characterization was performed on a CytoFLEX LX Flow Cytometer (Beckman Coulter) after calibration with fluorescent silica particles (0.1, 0.5, and 1.0 µm), and the EV gate was set below the 1 µm bead cloud as described in the Methods section. RBC-derived EVs (**b**), monocytic EVs (**c**) and MSC-derived EVs (**d**) were stained with annexin V as marker for EVs exposing phosphatidylserine, as well as with specific cell surface markers. Staining of EVs was performed as described in the methods section and a representative forward scatter *vs.* violet side scatter (FS *vs.* violet SS) density plot (left panel) as well as a representative annexin V vs. specific cell surface marker density plot (right panel) is shown for each EV type.

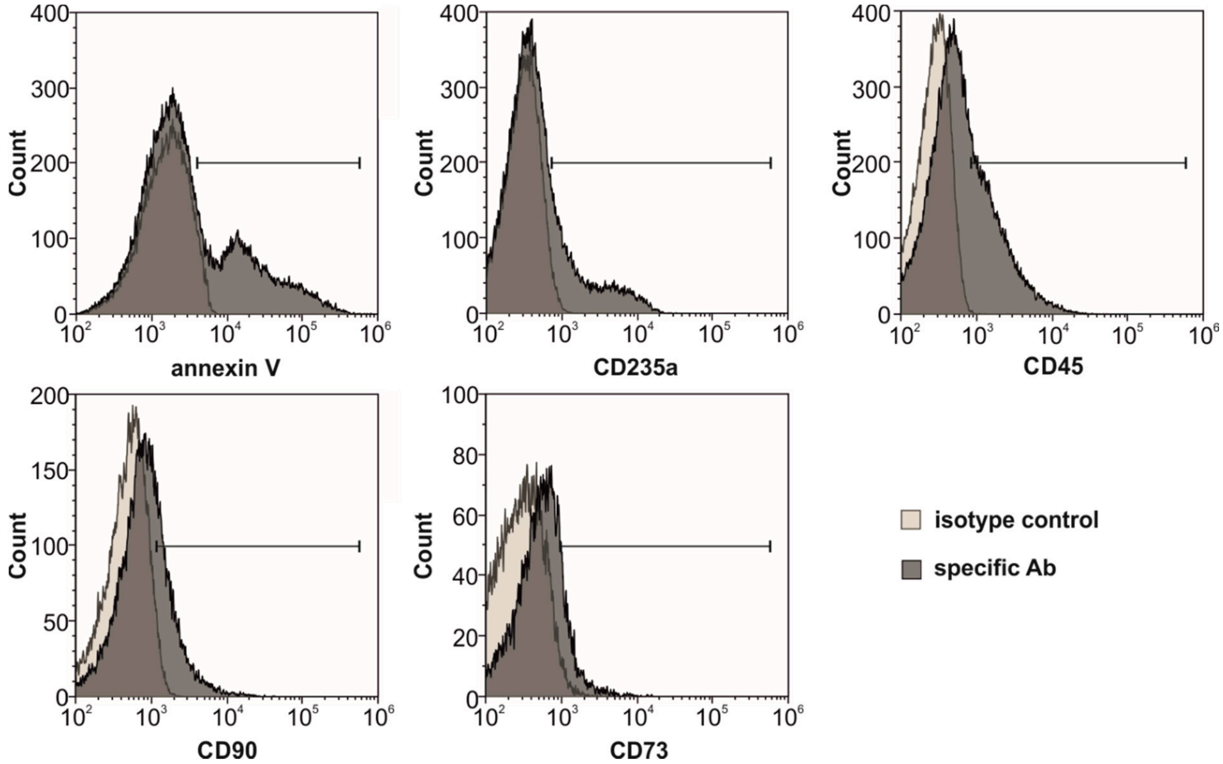

Figure S2. Controls for the flow cytometric characterization of EVs. The respective isotype control and single stainings are shown. Annexin V staining in PBS medium without $CaCl_2$ and $MgCl_2$ was used as negative control. Bars indicate positive expression.

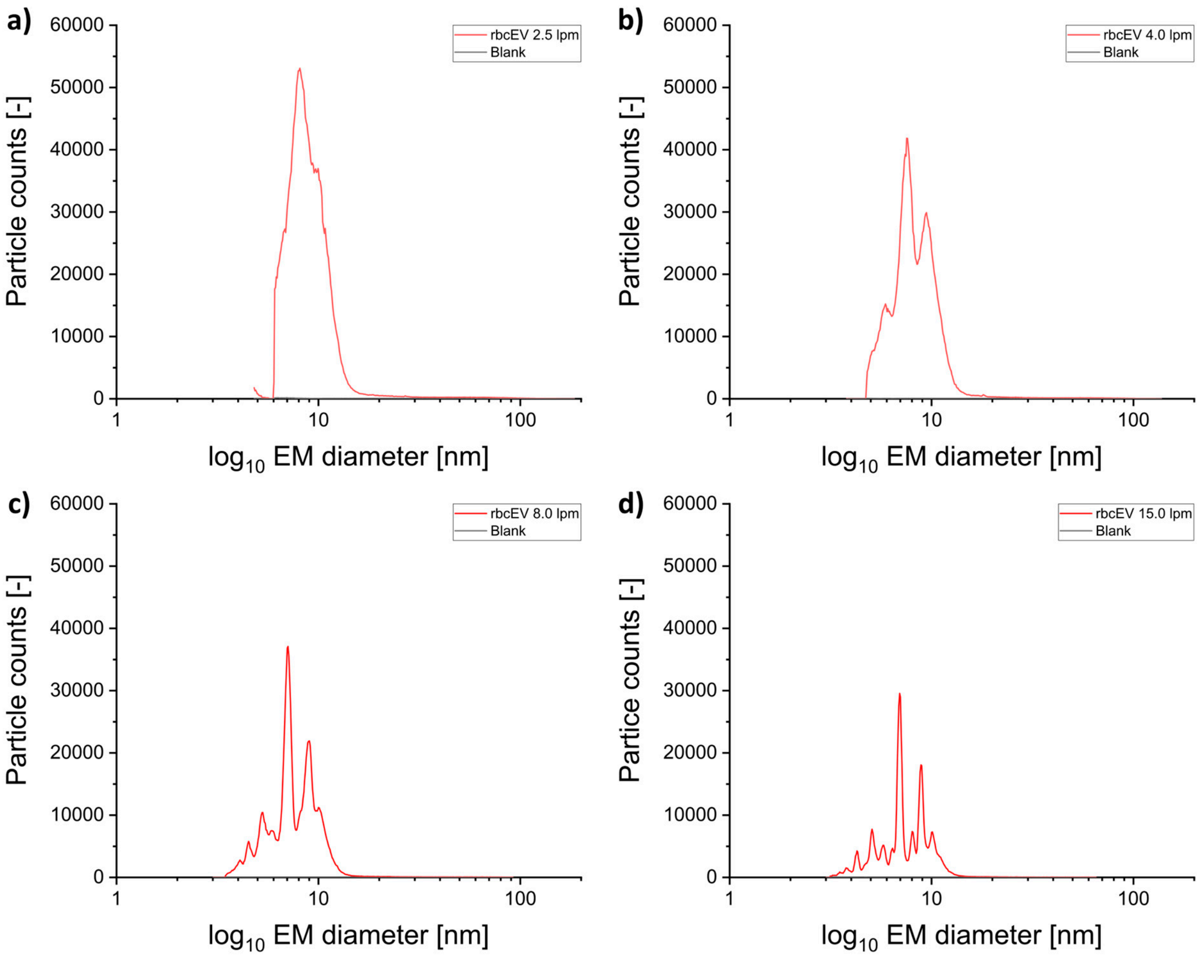

Figure S3. Effect of various sheath flow settings of nES GEMMA measurements on the separation of protein contaminants. The separation of complex protein mixtures is more efficient with higher sheath flow settings (15.0 L/min (**d**) and 8.0 L/min (**c**)), compared to lower settings (2.5 L/min (**a**) and 4.0 L/min (**b**)). For the characterization of EVs finally a sheath flow of 8.0 L/min (was employed).

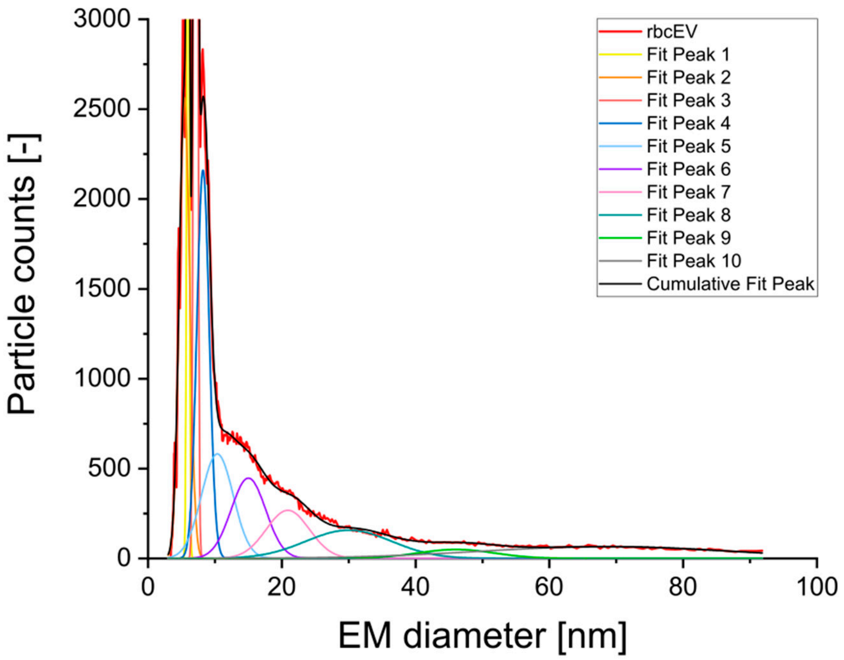

Figure S4. Fitting of Gaussian peaks to rbcEV spectra of nES GEMMA measurements resulting in peaks at 21 nm, 30 nm, 46 nm and 68 nm in the upper nm-range (≥ 20 nm "EV-range").

Table S1. Parameters of fitted Gaussian peaks of the rbcEV spectra of nES GEMMA measurements (Supplementary Figure 3) with y0 = 0 for all fitted peaks, resulting in R^2 = 0.9961.

	**xc**	**w**	**A**
**Fit Peak 1**	5.97 ± 0.00	0.36 ± 0.01	2116.90 ± 71.72
**Fit Peak 2**	5.45 ± 0.04	1.54 ± 0.07	4957.06 ± 570.74
**Fit Peak 3**	7.11 ± 0.00	0.50 ± 0.01	6023.66 ± 123.23
**Fit Peak 4**	8.21 ± 0.05	1.84 ± 0.16	4988.04 ± 1099.31
**Fit Peak 5**	10.35 ± 5.02	4.74 ± 4.34	3456.14 ± 9483.28
**Fit Peak 6**	15.00 ± 4.73	5.18 ± 12.07	2902.44 ± 15,304.71
**Fit Peak 7**	20.90 ± 9.06	6.46 ± 15.25	2166.54 ± 12,550.77
**Fit Peak 8**	30.00 ± 13.57	12.85 ± 44.35	2536.89 ± 10,227.03
**Fit Peak 9**	46.05 ± 27.06	11.53 ± 41.78	720.50 ± 5526.89
**Fit Peak 10**	68.49 ± 27.74	37.82 ± 68.54	3123.28 ± 5489.80
